# An Idealized Model of Extrusome Ejection Through the Collapse of a Capsule

**DOI:** 10.1007/s11538-026-01684-6

**Published:** 2026-06-17

**Authors:** Addie Harrison, Wanda Strychalski, Christina Hamlet, Laura Miller

**Affiliations:** 1 Department of Mathematics, University of Arizona, 617 N. Santa Rita Ave. P.O. Box 210089, Tucson, 85721-0089 AZ USA; 2 Department of Mathematics, Applied Mathematics and Statistics, Case Western Reserve University, 10900 Euclid Ave., Cleveland, 44106-7058 OH USA; 3 Department of Mathematics, Bucknell University, 359 Olin Science Building, Lewisburg, 17837 PA USA

**Keywords:** immersed boundary method, fluid dynamics, nematocyst, extrusome, ejection, cellular process

## Abstract

Extrusomes are extrusive organelles found in a variety of organisms, including cnidarians, dinoflagellates, and protists. These organelles typically contain a fluid-filled capsule that houses a structure, such as a coiled tubule or barb, which is rapidly ejected toward a target. Although the ejection mechanisms and morphology vary widely, a common feature is the rapid acceleration of the ejected structure through a fluid. In this paper, we develop an idealized model to simulate the collapse of an extrusome capsule, which enables the ejection of a barb or other internal structure. Specifically, we used the immersed boundary method to numerically simulate the collapse of a simplified capsule, modeled as the two sides of an elliptical shell with a flat plate along the bottom. As the capsule collapses, the elliptical sides straighten, ejecting both the internal fluid and the enclosed structure towards either free fluid or a flexible target. We investigate the effects of key model parameters, such as the size of the capsule opening. We also explore the role of the Reynolds number (*Re*), to consider the fluid dynamics across a range of regimes, from inertial-dominated flows relevant to some extrusome firings to viscous-dominated flows characteristic of cellular-scale processes. Our results demonstrate that decreasing the capsule opening gap size leads to increased firing velocity and shorter ejection times. Similarly, increasing the capsule’s minor axis reduces the time it takes the barb to reach the target as a larger volume of fluid is moved. The relationship between *Re* and the time to contact is nonmonotonic, but higher *Re* values generally result in faster target contact, even at longer initial distances. Furthermore, we observe that higher *Re* values enhance the robustness of the target contact in different configurations. Finally, we quantify how the stiffness of the barb affects its ability to reach the target. We find that the large deformations of the flexible barbs slow their trajectories and that the stiffer barbs hit their targets sooner. These findings provide a foundational understanding of the biomechanics and fluid dynamics of extrusome ejection through the collapse of a capsule. The insights gained may contribute to the development of microinjectors in drug delivery, where precise and rapid mechanical movements are critical.

## Introduction

Extrusomes are extrusive organelles of protists, cnidarians, and other organisms, released in response to specific stimuli (Rosati and Modeo 2003; Hausmann 1978). A notable type of extrusome is the nematocyst, which is utilized by members of the Cnidaria phylum and certain microorganisms, such as dinoflagellates (Mariscal 1974; Östman 2000). Nematocysts serve a variety of functions, such as capturing prey, defense, transport, and digestion. Although extrusomes generally function to deliver venom and other substances, nematocysts differ in their complex structure: a fluid-filled elastic capsule containing a highly coiled tubule tipped with a barb. The fluid that fills the capsule in the last stage of nematocyst development is a high concentration of poly-$\gamma$-glutamate, which is associated with generating an extreme osmotic pressure gradient due to the concentration of cations (Lotan 2016). Although commonly referred to as stinging cells, it is important to note that the nematocyst is the capsule that facilitates the delivery of venom during the stinging process (Fautin 2009).

**Fig. 1:**
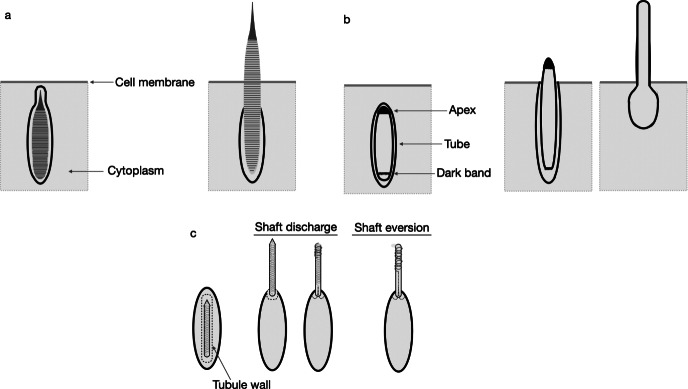
Diagram of different extrusome ejection mechanisms. a) *Paramecium* trichocyst ejects through mechanical stretching that results in rapid discharge (redrawn from Hausmann and Mignot (1975)). b) Protist rhabdocysts ejection occurs when swelling in the proximal end forces extrusion (redrawn from Hausmann (1978)). c) Anemone trichous nematocysts discharge when the shaft undergoes eversion (redrawn from Karabulut et al. (2022))

The specific ejection process varies widely across species and is still debated in cnidarians (Hausmann 1978; Watson and Mire-Thibodeaux 1994). Fig. 1 shows a schematic of some of the main ejection and firing mechanisms. For example, the protist rhabdocyst undergoes a telescopic explosion due to swelling in the proximal part of the extrusome, while flagellates ejectosomes involve the uncoiling of a ribbon (Hausmann 1978). Other means of ejection include paramecium trichocysts, which utilize a stretching mechanism to unfold a 3D network of protein filaments; stenotele nematocysts, which evaginate a tubule; and hydra stenoteles, which first evert three stylets that form an arrowhead and then evaginate while the stylets withdraw (Rosati and Modeo 2003; Hidaka 1993). More straightforward morphologies, such as the trichous and atrichous nematocyst, involve the initial ejection by a long tubule, followed by the inversion of the shaft and tubule after the discharge from the capsule (David et al. 2008; Karabulut et al. 2022; Rosati and Modeo 2003). A comprehensive overview of cnidocyst structure and discharge biomechanics is given in Özbek et al. (2009).

There are at least four general hypotheses about the mechanism behind nematocyst discharge: the contraction hypothesis, the tension hypothesis, the osmotic hypothesis, and the stopper hypothesis (Watson and Mire-Thibodeaux 1994). The simplest mechanism is the contraction hypothesis, whereby the capsule wall contracts (Cormier and Hessinger 1980). The basic idea behind the tension hypothesis is that the nematocyst capsule and/or tubule stores energy that is released during discharge. In the case of the osmotic hypothesis, the osmotic pressure of the nematocyst increases before discharge, and the capsule swells. During discharge, the volume of the capsule decreases. The stopper hypothesis works in combination with the other hypotheses, and the opening of the stopper allows the release of stored tension.

For all of the potential ejection mechanisms, the fluid dynamics shifts from the viscous-dominated regime to the inertial regime due to the extreme acceleration achieved by the firing process. We will use the Reynolds number *Re* to discuss the fluid regime. The *Re* is a dimensionless quantity used in fluid dynamics that is found by non-dimensionalizing the Navier-Stokes equations. Flows of the same *Re* are dynamically similar. The Reynolds number gives the ratio of inertial forces to viscous forces occurring within a fluid and is given as $Re= \rho L U/ \mu$, where $\rho$ is the density of the fluid, *L* is a characteristic length, *U* is a characteristic velocity, and $\mu$ is the dynamic viscosity of the fluid. In this case, *L* is the length of the barb, and *U* is the maximum velocity exerted during the contraction of the capsule, which we have calculated as the length of the barb over the period of cell contraction. The relevant *Re* range for nematocysts is from around $10^2$ to $10^3$ (Holstein and Tardent 1984), while the relevant *Re* for most cellular processes is much less than one.

Discharge has previously been modeled by applying an external force to accelerate the barb in a viscous fluid (Hamlet et al. 2020), and the case of multiple barbs fired together from separate capsules using the same force-driven approach has been examined in Harrison et al. (2025). It is known that physical deformations are part of the firing process (Lotan 2016; Nüchter et al. 2006), and here we include the effects of the mechanical process of the enclosing capsule’s deformation during firing in addition to the hydrodynamics. Previous mathematical models have focused on the role of chemical gradients during nematocyst discharge (Park et al. 2016) and general principles of power-amplifying mechanisms in biology (nematocyst discharge being one example of such a system) (Ilton et al. 2018). In Park et al. (2016), the authors studied nematocyst discharge through the build up of the poly-$\gamma$-glutamate osmotic potential and provided a differential equation model to describe nematocyst tubule elongation after firing. In Ilton et al. (2018), the authors explore how very small systems−both biological (like mantis shrimp, trap-jaw ants, and froghoppers) and engineered (like microrobots)-achieve extraordinarily fast movements. Their mathematical model consists of force balance using a simplified motor, spring, and latch models without hydrodynamics.

It is worth being clear about which extrusomes our collapsing-capsule model is intended to represent. The fluid-driven discharge we simulate is most consistent with extrusomes whose firing involves a net decrease in capsule volume, with the projectile carried passively in the resulting jet. Spindle trichocysts in *Paramecium* and *Frontonia* fit this picture. Their crystalline matrix rapidly decondenses and expands, and the surrounding capsule changes shape as the contents are released (Rosati and Modeo 2003; Hausmann 1978). The trichites of *Strombidium* and *Novistrombidium*, which we used to set the geometric parameters of the base case, also show capsule-shape change during discharge (Rosati and Modeo 2003; Modeo et al. 2001, 2003). Protist rhabdocysts, where swelling at the proximal end forces extrusion, share the same essential physics of a pressurized capsule emptying through a fixed opening (Hausmann 1978). The dinoflagellate “nematocysts” described by Gavelis et al. involve discharge from a pressurized capsule and are another natural target for this kind of model (Gavelis et al. 2017; Westfall et al. 1983). Among cnidarian nematocysts, our model is most relevant to those whose discharge fits the contraction or osmotic hypothesis, where the capsule volume decreases during firing (Watson and Mire-Thibodeaux 1994; Lotan 2016), rather than to discharge modes dominated by tubule eversion. By focusing on this shared mechanical class, we can ask fundamental physics questions, such as how fast the barb moves, whether it reaches the prey, and how the boundary layer behaves at different Reynolds numbers. This physics can be applied across all of these systems without committing to any one biochemical trigger.

In this study, we develop an idealized model of extrusome ejection where a fluid-filled capsule collapses and ejects the fluid and a flexible barb within it. This model is most consistent with the contraction and osmotic hypotheses, where the volume of the capsule is decreased during discharge. The fundamental difference in terms of the physics of discharge is that a fluid volume is propelled from the capsule, and the barb is passively transported with it, in contrast to an accelerating barb that drags viscous fluid along with it. This difference has consequences for the barb’s ability to hit a target since the volume of fluid can act to push the target out of the way.

This study focuses on understanding the individual ejection mechanics of extrusomes, particularly nematocysts, and how fluid dynamics, such as the Reynolds number (*Re*), impact the acceleration and travel distances of a discharged barb. We employ a 2D version of the immersed boundary method to simulate a flexible barb within a contracting capsule that has been ‘unstopped’ (i.e., there is an opening at the top of the capsule). The two sides of the capsule are constructed using half ellipses, the bottom of the capsule is a flat plate, and the top of the capsule is open. The contraction is modeled by continuously decreasing the length of the minor axes of the ellipse. The barb is modeled as a simple, flexible beam that was initialized in the center of the capsule.

We first consider the effect of *Re* on the distance traveled by the barb ejected out of the capsule. To quantify the ability of the barb to hit a target, we then include a flexible, passive prey one barb length away from the opening of the capsule. Based on previous work that shows a value of $Re \ge 10$ is necessary to reach the prey (Strychalski et al. 2018; Hamlet et al. 2020), we expect that at low *Re*, large fluid boundary layers generated by the barb may move the prey further away from the barb in this model. $Re \ge 10$ may be necessary to reach the prey.

Extrusomes vary in their physical dimensions, and we hypothesize that the geometry of the capsule affects the barb’s speed and ability to reach the prey. We increase the maximum extent of the minor axis of the capsule, which results in an increase of ejected fluid volume and curvature of the elliptical capsule wall. We predict that this change in the geometry results in an increase in the fluid and barbs’ speed due to an increase in the ejected fluid volume. If the same fluid volume is ejected through a smaller gap, the speed of the fluid is increased. We hypothesize the barb will reach the prey more quickly than in the case of a larger gap. We further expect the boundary layer interactions to affect the relationship between gap size and ejection times more strongly at lower *Re* based on previous work.

The barb in our model can be thought of as an idealization of a more complex structure in a nematocyst that contains a coiled tubule attached to a stylet (Nüchter et al. 2006; Gavelis et al. 2017). Biologically, the mechanical properties of the barb are nonuniform, and we consider this in our model by varying the flexural stiffness of the barb. We consider the cases of varying the flexural stiffness uniformly and then spatially along the barb. We hypothesize that a soft barb will be unable to maintain a straight shape and reach the prey after firing. If we consider a barb that is more stiff at the top (to mimic the stylet) connected to a softer region (to mimic the tubule), we expect the barb still to be able to reach the prey, albeit not as fast as a completely stiff barb.

## Methods

**Fig. 2:**
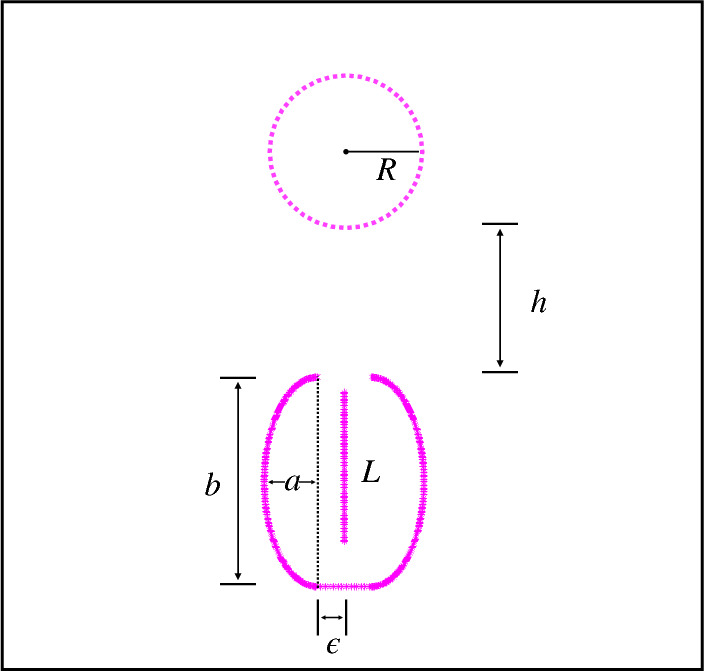
Schematic of the capsule, barb, and prey in the fluid-structure interaction model. A barb (magenta) ejects out of a flexible capsule (magenta) in a viscous fluid. The model can either be a prey present or no prey, where the prey (dashed magenta circle) has radius *R*.

We construct an idealized 2D model of a contracting capsule with an internal barb similar to the configuration of a trichocyst (Fig. 1a). The complete model schematic is shown in Fig. 2 and is described in detail here. The model consists of structures (barb, capsule, and prey) immersed in a viscous fluid. The barb is a rigid line of length *L* that is initially enclosed within the capsule. The capsule consists of flexible, elliptical side walls, where the ellipse has a major axis *b* and minor axis *a*.

The flat base connection consists of a horizontal line with length $2\epsilon$. The top end is left open to eject the barb.

When present, the prey is modeled as a passive, flexible circle with a radius of *R* that is positioned one barb length *L* away from the capsule’s opening.

Below, we describe the constitutive equations for the structures, the fluid equations, fluid-structure interactions, and the kinematics of the collapsing capsule. We also describe the numerical methods used to implement the model.

**Fig. 3:**
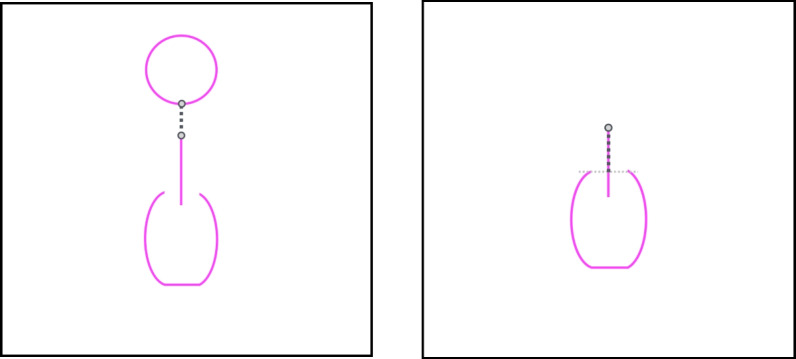
Schematic diagrams of the capsule, barb, and prey (solid magenta lines) as well as the distance reported in the methods (gray dashed line). Left: Simulations that consider the capsule, barb, and prey. The distance (gray dashed line) is calculated as the shortest distance from the tip of the barb (gray point) to the closest Lagrangian point on the prey (gray point). Right: A model of a capsule ejecting a barb in the absence of prey. Here, the distance reported is the distance between the barb’s tip (gray point) and the top of the capsule along its central axis.

### Constitutive Equations and Kinematics

The barb, capsule, and prey are each modeled as deformable elastic structures immersed in a viscous, incompressible fluid. The barb and prey experience elastic forces due to bending and stretching from a rest configuration,

1 $$\begin{aligned} {\textbf {F}}^{\text {st}}_{\text {elastic}} ={\textbf {F}}^{\text {st}}_{\text {spring}}+{\textbf {F}}^{\text {st}}_{\text {bend}}, \end{aligned}$$

where structure (st) denotes either the prey (p) or barb (b), ${\textbf {F}}^{\text {st}}_\text {spring}$ is the force density due to elasticity from stretching, and ${\textbf {F}}^{\text {st}}_\text {bend}$ is the elastic force density that arises from resisting bending. Forces are computed by taking derivatives of the elastic energy, and we describe the energies due to stretching and bending next.

For both the barb and the prey, adjacent Lagrangian points on a discretized structure are connected by linear springs with zero resting length. If the elastic link *l* connects two immersed boundary points ${\textbf {X}}^{\text {st}}_{p(l)}$ and ${\textbf {X}}^\text {st}_{q(l)}$, the elastic energy for this link is

2 $$\begin{aligned} E_S^{\, \text {st}}({\textbf {X}}^\text {st}_{p(l)}, {\textbf {X}}^\text {st}_{q(l)}) = \frac{k^{\text {st}}_{\text {spring}} }{2} \left( \left| {\textbf {X}}^{\text {st}}_{p(l)} - {\textbf {X}}^{\text {st}}_{q(l)} \right| -R^{\text {st}}_{l} \right] )^2, \end{aligned}$$

where $k^{\text {st}}_{\text {spring}}$ is the spring stiffness coefficient, and $R_l^{\text {st}} = 0$ is the rest length of the spring. Note that model parameter values are listed in Table 1.

To calculate the bending energy, we consider two links connecting points ${\textbf {X}}^{\text {st}}_{p}, {\textbf {X}}^{\text {st}}_{q},$ and ${\textbf {X}}^{\text {st}}_{r}$. The bending energy penalizes deviations in the angle between these links from a prescribed angle and is given by

3 $$\begin{aligned} E_B^{\, \text {st}}({\textbf {X}}^{\text {st}}_{p}, {\textbf {X}}^{\text {st}}_{q},{\textbf {X}}^{\text {st}}_{r}) = \frac{k_{\text {bend}}^{\text {st}}}{2} \left( {\textbf {k}} \cdot ({\textbf {X}}^{\text {st}}_{r} - {\textbf {X}}^{\text {st}}_{q}) \times \left( {\textbf {X}}^{\text {st}}_{q}- {\textbf {X}}^{\text {st}}_{p} \right) - C \right) ^2, \end{aligned}$$

where ${\textbf {k}} = (0,0,1)$, $k^{\text {st}}_{\text {bend}}$ is the bending stiffness coefficient, and *C* is a specified curvature value. The bending energy is minimized when the angle $\theta$ between the links satisfies

$$\begin{aligned} \sin \theta = \frac{C}{\left| {\textbf {X}}^{\text {st}}_{r} - {\textbf {X}}^{\text {st}}_{q} \right| \left| {\textbf {X}}^{\text {st}}_{q} - {\textbf {X}}^{\text {st}}_{p} \right| }. \end{aligned}$$

To drive the energy towards a specified angle $\psi$, the constant *C* should be set to the value

$$\begin{aligned} C = R_{pq}R_{qr} \sin \psi , \end{aligned}$$

where $R_{pq}$ and $R_{qr}$ are the resting lengths of the elastic links connecting points ${\textbf {X}}^{\text {st}}_{r}$ to ${\textbf {X}}^{\text {st}}_{q}$ and ${\textbf {X}}^{\text {st}}_{p}$ to ${\textbf {X}}^{\text {st}}_{q}$, respectively. In our model, we set $C=0$ to model zero preferred curvature.

The total elastic energy is the sum of the stretching and bending energies for each immersed boundary point in a structure,

4 $$\begin{aligned} E^{\, \text {st}}({\textbf {X}}^{\text {st}}_{1}, {\textbf {X}}^{\text {st}}_{2}, \dots , {\textbf {X}}^{\text {st}}_{N}) = \sum _{j=1}^{N - \delta } E_S^{\, \text {st}} ( {\textbf {X}}^{\text {st}}_{j},{\textbf {X}}^{\text {st}}_{j+1}) + \sum _{j=1+ \delta }^{N - \delta } E_B^{\, \text {st}} ( {\textbf {X}}^{\text {st}}_{j-1}, {\textbf {X}}^{\text {st}}_{j}, {\textbf {X}}^{\text {st}}_{j+1}), \end{aligned}$$

where *N* is the total number of points in the structure, $\delta =0$ if there is a spring connecting ${\textbf {X}}^{\text {st}}_{1}$ to ${\textbf {X}}^{\text {st}}_{N}$, and $\delta =1$ otherwise. The force density is calculated by computing

5 $$\begin{aligned} {\textbf {F}}_{\text {elastic}}^{\text {st}} = - \frac{ \partial E^{\, \text {st}}({\textbf {X}}^{\text {st}})}{\partial {\textbf {X}}^{\text {st}}_{i}}. \end{aligned}$$

Explicit formulas for the forces can be found in Battista et al. (2017).

The capsule’s force density consists of forces due to bending and target points used to prescribe motion,

6 $$\begin{aligned} {\textbf {F}}^{\text {c}} ={\textbf {F}}^{\text {c}}_{\text {bend}}+{\textbf {F}}^{\text {c}}_{\text {target}}. \end{aligned}$$

In the IB method, tether forces are used to prescribe the motion of an immersed structure. The capsule tether forces are given by

7 $$\begin{aligned} {\textbf {F}}^{\text {c}}_{\text {target}} = -k_\text {target} ({\textbf {X}}^{\text {c}} - {\textbf {Y}}^{\text {c}}), \end{aligned}$$

where ${\textbf {X}}^{\text {c}}$ is the position of a Lagrangian point and ${\textbf {Y}}^{\text {c}}$ is its associated target point (described below). The stiffness constant $k_\text {target}$ is chosen so that the displacement between each point and its target is no more than 1% of the total length of the barb.

The initial position of the capsule is shown in Fig. 2 along with the initial configuration of the capsule with two elliptical walls connected by a base of length $2\epsilon$. One side of a capsule wall is initialized by the parameterized curve

8 $$\begin{aligned} \textbf{X}_{\text {capsule}}(s,0)= (x_c \pm \epsilon ,y_c)+(a\cos (s),b\sin (s)), \quad \frac{\pi }{2} \le s \le 2 \pi + \frac{\pi }{2}, \end{aligned}$$

where $a = 0.2$, $b =0.7$, $(x_c,y_c) = (L/2,L/2-b)$, and $\pm \epsilon$ refers to either the $(-)$ left or $(+)$ right capsule wall.

During the collapse of the capsule, the preferred positions of the left and right sides of the capsule are updated. The the *x* component of ${\textbf {Y}}^{\text {c}}$, denoted as $X^c(s,t)$ below, is updated according to the equation,

9 $$\begin{aligned} X^c(s,t) = x_c + a \left[ 1-\left( \frac{t}{t_{\text {period}}} \right) \right] \cos (s) \pm \epsilon , \quad \frac{\pi }{2} \le s \le 2 \pi + \frac{\pi }{2}, \end{aligned}$$

where $\pm \epsilon$ refers to either the $(-)$ left or $(+)$ right capsule wall. The *y*-component remains the same as its initial value.

The specified motion of the capsule walls results in decreasing the curvature of the ellipse so that the walls are close to vertical lines at the end of the collapse.

### Fluid-Struction Interaction Equations and the Immersed Boundary Method

The barb, capsule, and prey are immersed in a viscous, incompressible fluid modeled by the Navier-Stokes equations,

10 $$\begin{aligned} \rho (\textbf{u}_t(\textbf{x},t)+\textbf{u}(\textbf{x},t)\cdot \nabla \textbf{u}(\textbf{x},t))=-\nabla p(\textbf{x},t)+\mu \nabla ^2\textbf{u}(\textbf{x},t)+\textbf{f}(\textbf{x},t). \end{aligned}$$

11 $$\begin{aligned} \nabla \cdot \textbf{u}(\textbf{x},t)=0, \end{aligned}$$

where $\rho$ is the fluid density, $\mu$ is the fluid viscosity, $\textbf{x}=(x,y)$ are fixed Cartesian coordinates, *t* is the time, *p* is the fluid pressure, $\textbf{u}$ is the velocity field, and $\textbf{f}$ is the force per unit area applied to the fluid by the structure (described in Section 2.1).

The model is implemented in an immersed boundary (IB) framework (Peskin 2002). The fluid velocity $\textbf{u}$, pressure *p*, and force density $\textbf{f}$ are described in fixed, Eulerian coordinates, while the immersed structures (barb, prey, and capsule) are described in a Lagrangian framework.

The interaction between the Eulerian and Lagrangian frameworks is given by

12 $$\begin{aligned} \textbf{f}(\textbf{x},t)=\int _\Gamma \textbf{F}(q,t)\delta (\textbf{x}-\textbf{X}(q,t))\, dq, \end{aligned}$$

13 $$\begin{aligned} \textbf{X}_t(q,t)=\textbf{U}(\textbf{X}(q,t))=\int _\Omega \textbf{u}(\textbf{x},t)\delta (\textbf{x}-\textbf{X}(q,t))d\textbf{x} \end{aligned}$$

where *q* is the Lagrangian coordinate, $\textbf{F}$ is the elastic force density generated by deformations of the structure, $\delta (\textbf{x})$ is the 2D Dirac delta function, $\textbf{X}(q,t)$ denotes the parameterization of a the structure at time *t*, $\Gamma$ is the curve of the structure, and $\Omega$ is the fluid domain. Eq. (12) is the spreading operator that maps the force density from the Lagrangian framework to an Eulerian grid. The right-hand side of Eq. (13) interpolates the fluid velocity $\textbf{u}$ onto the velocity of the structure, $\textbf{U}$. Moving the structure at the local velocity enforces the no-slip boundary condition for the immersed Lagrangian structure.

### Nondimensionalization

To compare capsule contraction dynamics across a range of Reynolds numbers, we nondimensionalize the governing equations. To determine the relevant dimensionless parameters, we first set the appropriate dimensional quantities. The dimensional dynamic viscosity, $\mu$, and density, $\rho$, match those of seawater and are set to $1.08 \times 10^{-3}$ kg/(m $\cdot$ s) and 1025 $\text {kg/m}^3$, respectively. The characteristic length scale is chosen to be the barb length, approximately $L = 50$ microns, and the characteristic time scale is estimated as $T = 2.37 \times 10^{-6}$ seconds, which is the approximate duration of capsule collapse. The characteristic velocity is then given by $U = L/T=6.25$ m/s.

The incompressible Navier–Stokes equations in two dimensions can be written in dimensionless form as

14 $$\begin{aligned} \frac{\partial \textbf{u}'}{\partial t'} + \textbf{u}' \cdot \nabla ' \textbf{u}' = -\nabla ' p' + \frac{1}{Re} \nabla '^2 \textbf{u}' + \textbf{f}', \end{aligned}$$

15 $$\begin{aligned} \nabla ' \cdot \textbf{u}' = 0, \end{aligned}$$

where the Reynolds number is defined as

16 $$\begin{aligned} Re = \frac{\rho U L}{\mu }. \end{aligned}$$

The dimensionless variables are defined as

17 $$\begin{aligned} \begin{array}{ll} \textbf{u}' = \textbf{u} / U, & \quad t' = t U / L, \\ \textbf{x}' = \textbf{x} / L, & \quad p' = p / (\rho U^2), \\ \textbf{f}' = \textbf{f} / (\rho U^2 L), & \quad \nabla ' = L \nabla ,\\ \nabla '^2 = L^2 \nabla ^2. \end{array} \end{aligned}$$

Using the dimensional quantities defined above, the approximate *Re* for capsule ejection is about 1000. To consider a lower *Re* in the real system, one would increase the characteristic time scale, e.g., the duration of capsule collapse. The nondimensionalization allows us to easily compare the trajectories of the barb across these *Re* values.

The material parameters of the elastic structures must also be nondimensionalized. The spring stiffness coefficient $k_\text {spring}$ (with units of force per unit length) is nondimensionalized as

18 $$\begin{aligned} k'_\text {spring} = \frac{k_\text {spring}}{\rho U^2 L}. \end{aligned}$$

The bending stiffness coefficient $k_\text {bend}$ (with units of force times length) is nondimensionalized as

19 $$\begin{aligned} k'_\text {bend} = \frac{k_\text {bend}}{\rho U^2 L^3}. \end{aligned}$$

Table 1 shows the dimensionless quantities used in the simulations.

**Table 1:** Dimensionless parameters values for the capsule ejection model.

Parameter	Value
Characteristic length (Length of barb) (*L*)	1
Distance between capsule and prey (*h*)	1-2
Capsule sides minor axis (*a*)	0.08 - 0.24
Capsule sides major axis (*b*)	0.7
Capsule base ($\epsilon$)	0.08 - 0.14
Radius of prey (*R*)	0.3
Fluid domain ($\Omega$)	10 $\times$ 10
Time step ($\Delta t$)	$5\times 10^{-5}$
Spatial step ($\Delta x$)	0.0098
Eulerian grid pts. ($N_x$/$N_y$)	512
Reynolds number (*Re*)*	$0.029-1\times 10^{3}$
Characteristic velocity (*V*)	1
Spring stiffness ($k'_{\text {spring}}$)	$1\times 10^{13}$
Target stiffness ($k'_{\text {target}}$)	$1\times 10^{12}$
Beam stiffness ($k'_{\text {beam}}$)	$3.12\times 10^{16}$

### Numerical Discretization and IB2d

The nondimensional fluid domain size $\Omega$ is $10 \times 10$ with Eulerian grid spacing $\Delta x'= 10/512 \approx 0.0195$. The barb is discretized with a spacing of $\Delta s=L/1024=1/1024$, which allows for approximately two Lagrangian points per grid cell. In our simulations, the barb contains 103 points. The parameterization of the capsule edges is given in Eq. (8). Using our value of $\Delta s$, each side contains 225 points. The bottom plate that connects the ellipse is discretized with a spacing of $\Delta s=L/1024$ and contains 20 points. The prey is parametrized as a circle

20 $$\begin{aligned} \textbf{X}_{\text {prey}}(s,0)= (0,R)+R(\cos (s/R),\sin (s/R)), \quad 0\le s < 2\pi R, \end{aligned}$$

which contains 193 points.

We implemented the model using IB2d software in MATLAB (Battista et al. 2017). IB2d is an open-source fluid-structure interaction software that numerically implements the immersed boundary method in two spatial dimensions with robust options for artificial forcing. The numerical methods implemented in IB2d are formally second-order accurate in time and space.

## Results

We perform simulations either with prey (dotted circle in Fig. 2) or no prey. We first simulate two base cases at $Re=1000$ with and without a prey structure. We then vary the *Re* to model both viscous and inertial-dominated fluid regimes. To assess the significance of capsule modeling choices, we adjust both the capsule shape and the amount of collapse during the ejection process. We then study how the size of the capsule’s opening affects the ejection velocity and distance traveled by the barb. We simulate barb ejection with several values of the capsule opening gap length while holding all other parameters constant. Finally, we vary the barb’s stiffness coefficient to investigate how the barb’s flexibility influences its ability to make successful contact with the prey. For each of the cases considered, we present the location of the barb, capsule, and prey over time as well as the velocity magnitude and vorticity of the flow fields. We also present the distance traveled and/or the distance between the tip of the barb and the prey. In the absence of prey, we report the net distance traveled by the tip of the barb, measured as the distance from the largest *y*-value on the barb and the center point at the top of the capsule. In the presence of prey, we report the distance between the tip of the barb and the prey by calculating the distance between the barb tip (the largest *y* value Lagrangian point) and the closest point on the prey (see Fig. 3).

### Base Case

Here we present the base cases, both prey-present and no-prey models, with $Re=1000$. In both cases, the barb is positioned inside a capsule that has elliptical sides with the major axis set to $a=0.2$ and the minor axis set to $b=0.7$. The two sides are separated by a flat plate of length $2\epsilon$ (see Fig. 2). These relative length scales were chosen based on a longitudinal section of the trichites of *Novistrombidium testaceum* (Rosati and Modeo 2003). When prey is present, it is initially positioned $h=1$ away from the ejection site and centered above the barb. The radius of the prey is set to $R=0.3$ and the barb length is set to $L=1$. The capsule collapses over the characteristic length of time.

**Fig. 4:**
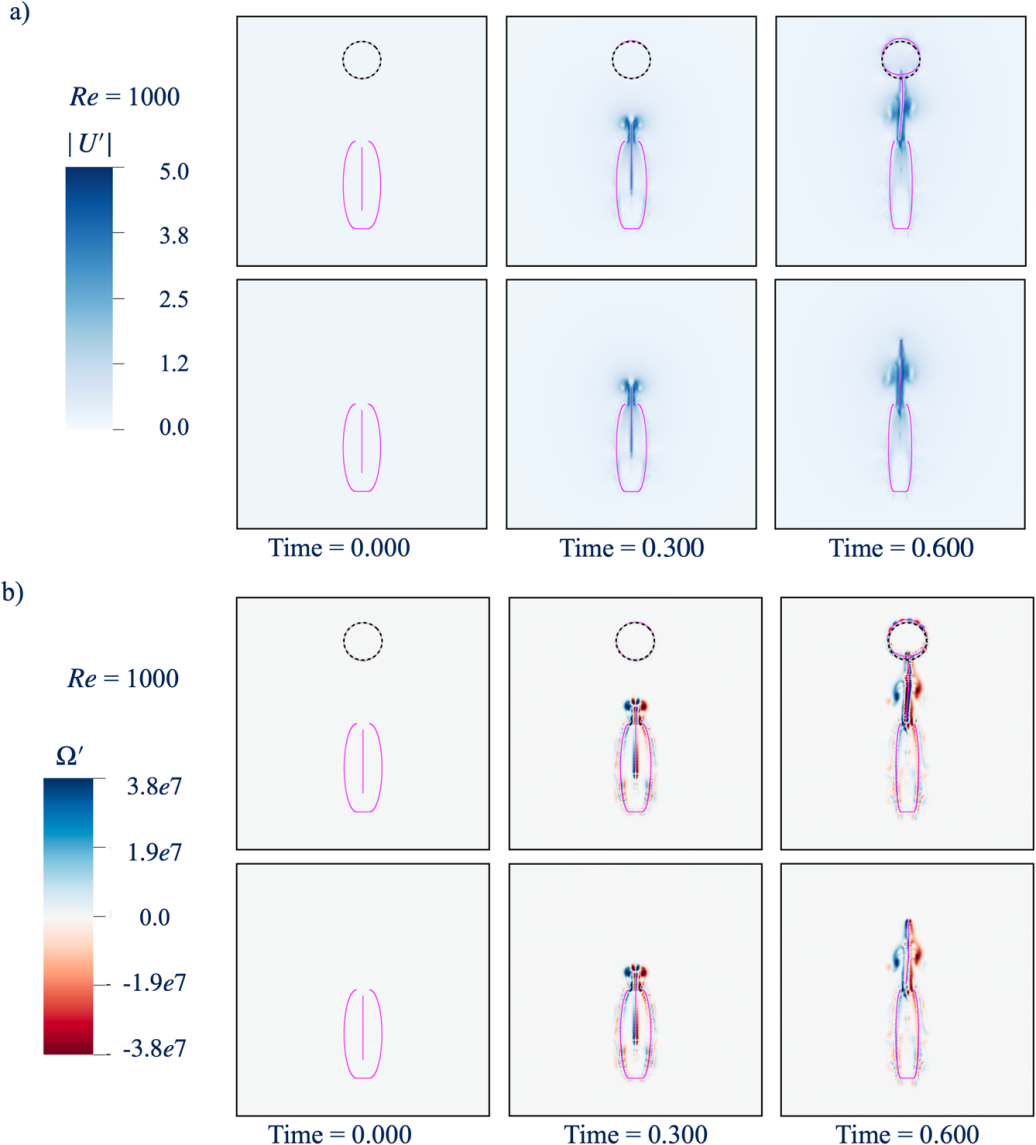
(a) Dimensionless velocity magnitude snapshots and (b) dimensionless vorticity magnitude snapshots for the base case with and without a prey. The barb, capsule, and prey (when present) are shown in magenta. The black dashed circle indicates the starting position of the prey at $t'=0$. The velocity magnitude is represented by a color map ranging from light blue to navy, while the vorticity magnitude ranges from dark red to navy. The simulations display snapshots at three dimensionless times, $t'=0$, $t'=0.3$, and $t'=0.6$. The base case employs a Reynolds number of 1000, a gap length of $2\epsilon = 0.200$, a minor ellipse axis of $a=0.2$, and a barb flexural stiffness of $3.13\times 10^{16}$.

Fig. 4 shows the velocity magnitude and vorticity of the prey-present and no-prey base cases at three different times: $t=0$, $t=0.3$, and $t=0.6$. The collapse of the capsule structure pressurizes the internal fluid, which pushes the barb and the internal fluid outside of the capsule. For both models at $t=0$, the barb and the fluid are at rest, and the capsule has not experienced the application of any collapsing force. Once the simulation begins, the internal fluid, along with the barb, is ejected from the capsule (see the middle images in both rows). In addition, oppositely spinning vortices are formed at the tip of the barb (middle column) and then separate and move downstream (right column). The final images at $t=0.6$ show that the barb has made contact with the prey if it was present. Note that the final time was selected as the instant when the barb reaches the prey. This distance is defined to be when the barb is within $4\Delta x$ of the prey, given the effective thickness of the boundaries in the IB method (Peskin 2002; Hamlet et al. 2020).

### Reynolds Number

**Fig. 5:**
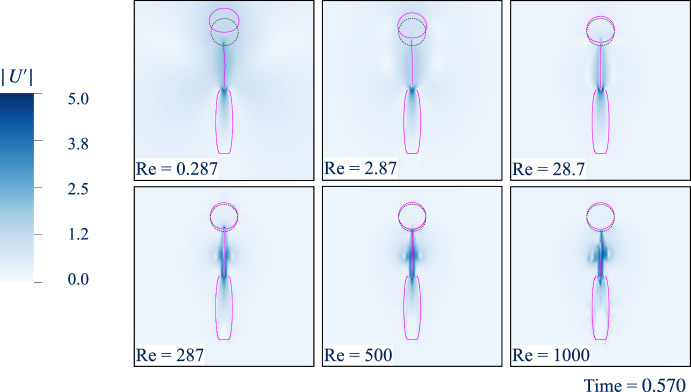
Dimensionless velocity magnitude snapshots for the ejection of a single barb from a capsule towards a circular prey with *Re* set to 0.287, 2.87, 28.7, 287, 500, and 1000. The capsule, barb, and prey are shown in magenta, and the black dashed circle shows the starting position of the prey. The magnitude of velocity in the fluid is represented by a color map, with the range shifting from light blue to navy. The simulation displays snapshots at a single time, $t'=0.57$. The initial distance between the capsule and the prey is set to $h=1$. Each simulation uses a gap length of $2\epsilon = 0.2$, a minor ellipse axis of $a=0.2$, and a barb coefficient stiffness of $3.13\times 10^{16}$.

To investigate the fluid dynamics across scales relevant to cellular processes, we varied *Re* from 0.287 to 1000. All other parameters remained unchanged as described in the base case and detailed in Table 1. Fig. 5 shows the dimensionless velocity magnitude for *Re* set to 0.287, 2.87, 28.7, 287, 500,  and 1000. This range covered the *Re* relevant to typical cellular processes up to the inertial-dominated flows characteristic of nematocysts. Each image shows the position of the structures and the magnitude of velocity at $t'=0.57$. The original prey position is shown as a dashed black circle. Note that the capsule collapses with a prescribed motion, detailed in the methods section. Also note that the displacement of the prey is significant for $Re<15$.

**Fig. 6:**
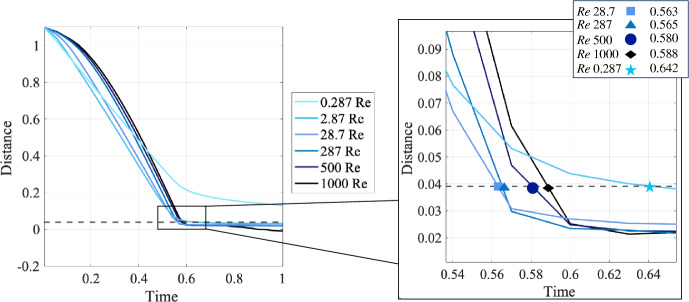
The dimensionless distance from the tip of the barb to the nearest point on the prey as a function of dimensionless time for *Re* set to 0.287, 2.87, 28.7, 287, 500,  and 1000. The initial distance between the barb and prey is set to $h=1$. The dashed black line denotes the threshold value for the contact of $4\Delta x$. The inset shows a zoomed-in view of the hit times or the higher *Re* simulations, and the legend highlights the specific time of each hit.

Fig. 6 shows the dimensionless distance between the top of the barb and the prey as a function of dimensionless time for the different *Re* considered. The inset of Fig. 6 allows one to see the precise time of contact for the five *Re* where contact with the prey is achieved (i.e., the barb is within $4\Delta x$ of the prey), and those times are given in the legend. At higher *Re* values ($Re \ge 287$), the velocity field is concentrated around the barb. The resulting contact times with the prey are $t'=0.565$ ($Re=287$), $t'=0.580$ ($Re=500$), and $t'=0.588$ ($Re=1000$). Note that in all cases, the barbs do reach the initial position of the prey (black dashed line); however, at lower *Re*, the larger boundary layers cause the flow speed farther from the barb to be higher. As a result, the velocity of the barb *relative to the prey* is slower. In the most viscous case ($Re=0.287$), the dimensionless distance between the barb and prey levels off around 0.130. Overall, the simulations reveal that higher Reynolds numbers reduce the degree to which the prey is pushed out of the way of the barb, allowing for faster contact.

**Fig. 7:**
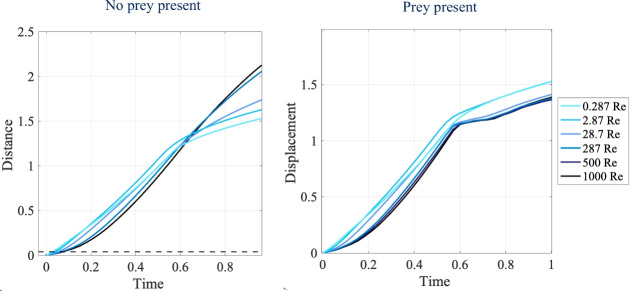
The dimensionless distance the barb has traveled with no prey present (left) and with prey present (right) for *Re* set to 0.287, 2.87, 28.7, 287, 500,  and 1000. The initial distance between the barb and prey, when present, is set to $h=1$.

Fig. 7 shows the dimensionless distance traveled by the barb with and without prey for each of the *Re* considered. When no prey is present, the barbs operating at lower *Re* initially travel faster than those at higher *Re*. After about $t'=0.6$, the lower *Re* barbs decelerate relative to the higher *Re* cases. When the prey is present, the higher *Re* barbs decelerate upon contact with the prey (around $t'=0.6$). The lower *Re* barbs decelerate more slowly as the prey is pushed out of the way.

**Fig. 8:**
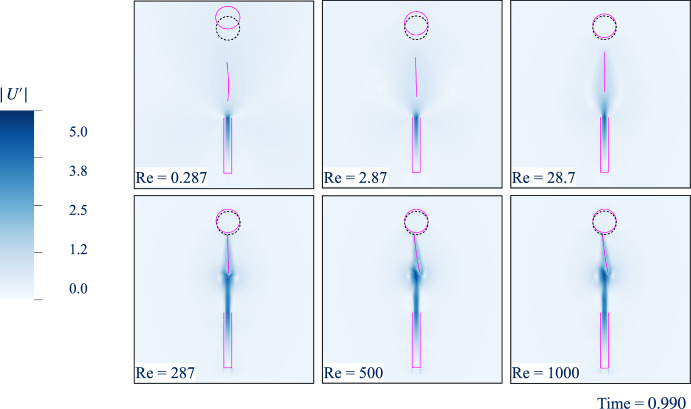
Dimensionless velocity magnitude snapshots for the ejection of a single barb from a capsule towards a circular prey with *Re* set to 0.287, 2.87, 28.7, 287, 500, and 1000. The capsule, barb, and prey are shown in magenta, and the black dashed circle shows the starting position of the prey. The dimensionless magnitude of velocity in the fluid is represented by a color map, with the range shifting from light blue to navy. The simulation displays snapshots at a single time, $t'=0.99$. The initial distance between the capsule and the prey is set to $h=2$. Each simulation uses a gap length of $2\epsilon = 0.2$, a minor ellipse axis of $a=0.2$, and a barb coefficient stiffness of $3.13\times 10^{16}$.

**Fig. 9:**
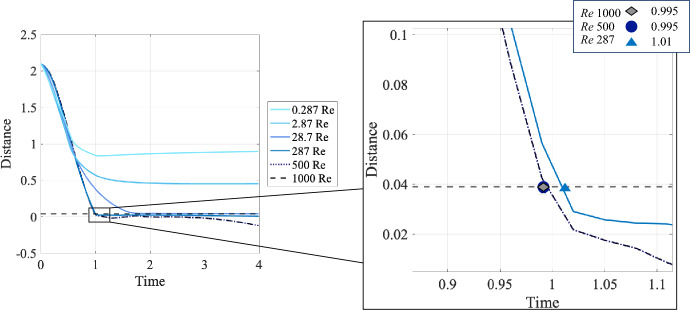
The dimensionless distance from the tip of the barb to the nearest point on the prey as a function of dimensionless time for *Re* set to 0.287, 2.87, 28.7, 287, 500,  and 1000. Note the distances for $Re=500$ and 1000 are the same. Therefore, we use dotted and dashed lines to highlight this. The initial distance between the barb and prey is set to $h=2$. The horizontal dashed black line denotes the threshold value for the contact of $4\Delta x$. The inset shows a zoomed-in view of the hit times or the higher *Re* simulations, and the legend highlights the specific time of each hit, where we use grey outlined in black for $Re=1000$ for color contrast of overlapping shapes.

To determine the effect of the initial position of the prey relative to the capsule, we performed a subsequent set of simulations for the same range of *Re* but with an initial distance between the capsule and the prey set to twice the original distance ($h=2$). Fig. 8 shows the velocity magnitude for *Re* set to 0.287, 2.87, 28.7, 287, 500,  and 1000. Similar to the simulations where the prey was closer to the capsule, the ejected fluid and barb significantly push the prey away from its initial position (dashed black line) for $Re \le 15$. We also see that the velocity field is more concentrated around the barb for higher *Re*, and the displacement of the prey is minimal. Fig. 9 shows the dimensionless distance from the tip of the barb to the closest point on the prey as a function of time when the initial distance between the capsule and prey is set to $h=2$. Note that for this larger initial distance, the barb does not hit the prey for $Re \le 2.87$. The inset shows the dimensionless times when the barb contacts the prey for larger *Re*: $t'=0.995$ for $Re=500$ and 1000, and $t'=1.00$ for $Re=287$. The overall trends for larger initial distances are more pronounced than for closer distances, as the larger gap exacerbates the effects of a diffuse velocity field that pushes the prey out of the way of the barb.

### Percent Volume Change in the Capsule

**Fig. 10:**
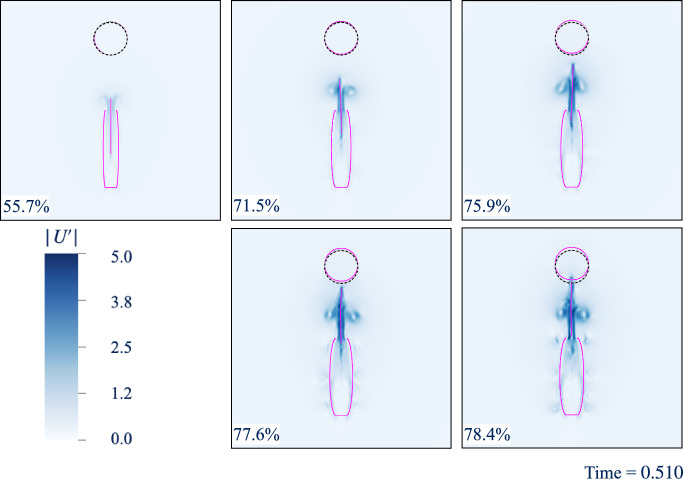
Dimensionless velocity magnitude graphs for simulations where the initial minor axis length is set to $a=0.08$, 0.16, 0.2, 0.22, and 0.24, corresponding to percent volume changes of 55.7%, 71.5%, 75.9%, 77.6%, and 78.4%, respectively. Each snapshot was taken at $t'=0.6$. The barb, capsule, and prey are represented in magenta, and the original prey position is shown as a black dashed circle. The magnitude of velocity is represented as a color map ranging from light blue to navy.

To determine how the percent volume change during capsule collapse affects the movement of the barb and contact with the prey, we vary the maximum extent of the minor axis of the capsule. Simulations were performed for minor axis lengths set to $a=0.08$, 0.16, 0.2, 0.22, and 0.24. These values for the minor axis correspond to changes in volume during the contraction of 55.7%, 71.5%, 75.9%, 77.6%, and 78.4%, respectively. Fig. 10 shows the barb position at $t'=0.51$. The percent volume change of the capsule is calculated by dividing the difference between the initial and final capsule area by the initial capsule area. The initial starting area is the area of the ellipse plus the area of the rectangle of dimensions $2\epsilon \times b$. The final capsule area is simply the area of a rectangle with width $2\epsilon$ and height *b*. For the largest initial minor axis, with $78.4\%$ volume change, we see regions of higher speeds surrounding the barb, due to the larger amount of fluid being removed from the capsule. For the case when the initial minor axis is the smallest, with a percent volume change of $55.7\%$, the barb’s movement out of the capsule is slower. Furthermore, the surrounding fluid has lower speed, and the barb does not come close to the prey.

**Fig. 11:**
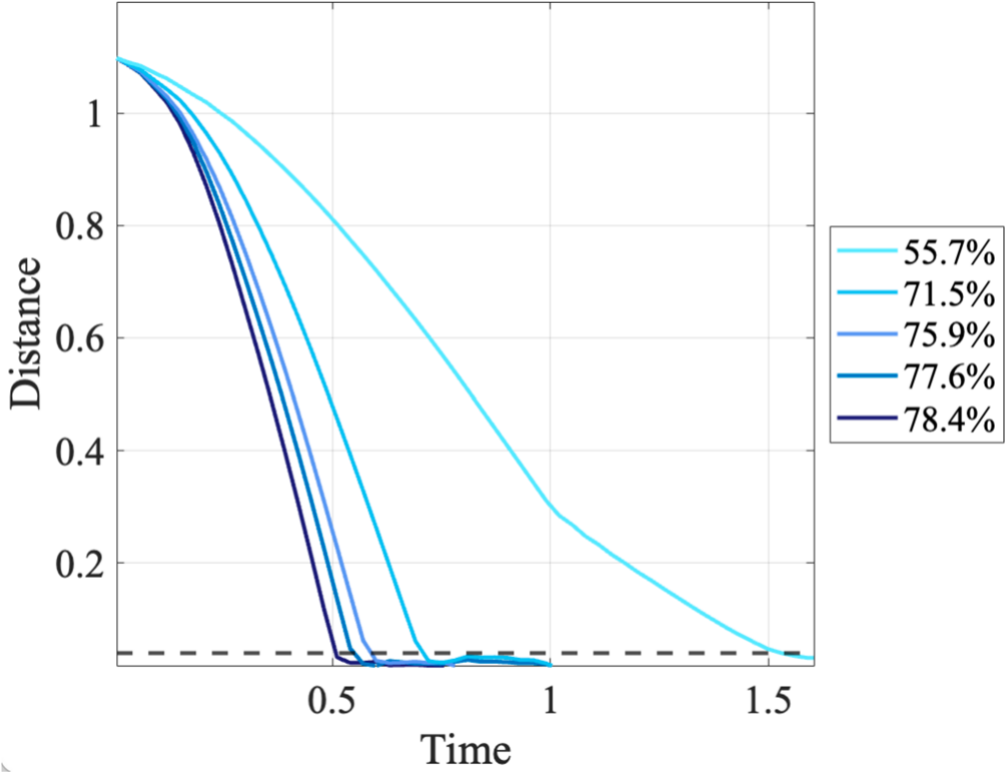
The dimensionless distance between the tip of the barb and the closest point on the prey as a function of dimensionless time for simulations where the initial minor axis length is set to $a=0.0800$, 0.160, 0.200, 0.220, and 0.240. Note that these values correspond to percent volume changes of 55.7%, 71.5%, 75.9%, 77.6%, and 78.4%, respectively. Each initial minor axis value is labeled from light blue to navy. The dashed line denotes the threshold value for the contact of $4\Delta x$.

Fig. 11 shows the dimensionless distance over dimensionless time between the barb and the prey, similar to previous graphs. As the initial minor axis length increases and the volume change is the largest, the distance between the barb and the prey decreases more rapidly. The overall time to hit the prey decreases as the volume of the capsule increases, suggesting that larger initial capsule volumes lead to shorter times to hit the prey.

### Gap Length

**Fig. 12:**
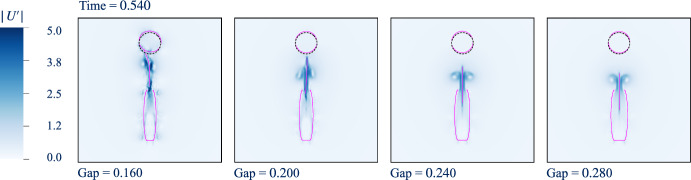
Dimensionless velocity magnitudes generated by capsule collapse when the gap length is set to 0.16, 0.2, 0.24, and 0.28. Each snapshot is taken at $t'=0.54$. The barb, capsule, and prey are represented in magenta, and the original prey position is shown in a black dashed circle. The magnitude of velocity is represented as a color map ranging from light blue to navy.

To investigate the effect of the size of the gap in the capsule where the barb exits, simulations were performed for gap lengths ($2 \epsilon$) of 0.16, 0.2, 0.24, and 0.28. The gap size was altered by adjusting the size of the plate at the bottom of the capsule, $\epsilon$. We note that the volume of fluid in the capsule increases with the gap length, but the volume of ejected fluid, i.e., the area of the ellipse $ab\pi$, remains the same as the gap size varies. Fig. 12 shows the dimensionless magnitude of velocity with a colormap from light blue to navy. The figure is taken at $t'=0.54$, which represents the fastest dimensionless time for the barb to hit the prey across all simulations when $2\epsilon =0.16$. As the gap size increases, the velocity of the barb decreases. A smaller gap size leads to higher velocities through the gap, as the same volume of ejected fluid must exit the capsule in the same amount of time. The increased fluid speed propels the barb through the capsule’s exit more rapidly, resulting in a shorter time to hit the prey. Importantly, the fluid velocity is sufficient to overcome any resistance from the narrow exit.

**Fig. 13:**
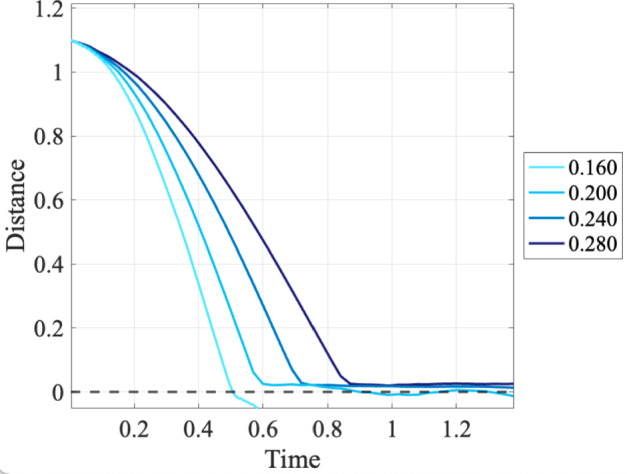
The dimensionless distance between the tip of the barb and the closest point on the prey vs. dimensionless time for gap lengths set to 0.16, 0.2, 0.24, and 0.28. Each gap length is labeled from light blue to navy. The dashed line denotes the threshold value for the contact of $4\Delta x$.

Fig. 13 shows the shortest dimensionless distance between the barb and prey for gap lengths of 0.16, 0.2, 0.24, and 0.28. The barb reaches the prey in all of the simulations. The trend is nearly linear, and as the gap size decreases, the time to hit the prey also decreases. In summary, the smaller gap size creates strong fluid acceleration through this constriction, leading to shorter barb ejection times. The shorter ejection times are a direct consequence of the interplay between the fluid dynamics and the system’s geometric constraints. The smaller the gap, the higher the fluid exit velocity, which translates into faster movement of the barb toward the prey.

### Barb Stiffness

**Fig. 14:**
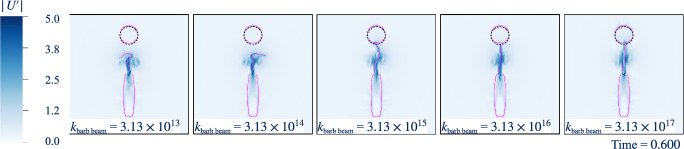
Dimensionless velocity magnitudes for barbs of different stiffnesses taken at $t'=0.6$. Note that $k'_\text {beam}$ is set to $3.13\times 10^{13},$ $3.13\times 10^{14},$ $3.13\times 10^{15},$ $3.13\times 10^{16},$ and $3.13\times 10^{17}$. The magenta outlines represent the capsule, barb, and prey positions, with the initial prey location denoted by a dashed black circle. The color map shows the magnitude of velocity, ranging from light blue (low velocity) to navy (high velocity).

To determine the effects of the barb’s bending stiffness on the ejection dynamics, we set $k'_\text {beam}= 3.13\times 10^{13},$ $3.13\times 10^{14},$ $3.13\times 10^{15},$ $3.13\times 10^{16},$ and $3.13\times 10^{17}$. Similar to the base case, we set $Re=1000$ and $a=0.2$. Fig. 14 shows the resulting dimensionless velocity color maps, structures (magenta), and starting prey position (dashed black circle) at $t'=0.6$. Once the stiffness is lowered to $k'_\text {beam}= 3.13\times 10^{13}$ and $3.13\times 10^{14}$, the barb became very flexible and did not move through the fluid in a straight path. This resulted in no contact with the prey at $t'=0.6$. Note that even if the barb continued to move toward the prey, the magnitude of force with which it hit the prey would be significantly lower, reducing its ability to puncture the prey. We also see that strong flow is not as localized around the barb. At an intermediate value, $k'_\text {beam}= 3.13\times 10^{15}$, the barb successfully reached prey, but the increased flexibility allows for deformations that could impact its ability to puncture the prey. At the larger values of $k'_\text {beam}$, the barb successfully reaches the prey and is less deformed. The largest value $k'_\text {beam}$ also shows the strongest localized flow around it, as can be seen from the thin layer around the magenta barb.

**Fig. 15:**
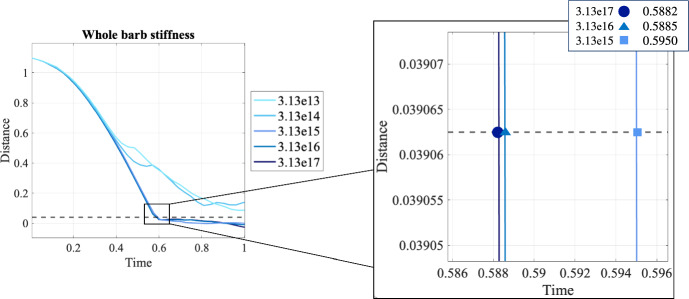
Dimensionless distance between the tip of the barb and the closest point on the prey as functions of dimensionless time for barb stiffnesses set to $k'_\text {beam}=$ $3.13\times 10^{13},$ $3.13\times 10^{14},$ $3.13\times 10^{15},$ $3.13\times 10^{16},$ and $3.13\times 10^{17}$. The dashed line denotes the threshold value for the contact of $4\Delta x$. The inset shows the time to hit for the three highest barb stiffness values.

The dimensionless distance between the tip of the barb and the closest point on the prey is shown in Fig. 15. For $k'_\text {beam}= 3.13\times 10^{13}$ and $3.13\times 10^{14}$ at $t'_\text {final}=1.00$, we see that the barb does not reach the prey. The barbs also considerably slow down upon exit from the capsule, as seen at $t'=0.540$. Note that at this point, the barb begins to deform significantly. In comparison, the three stiffer barbs with $k'_\text {beam}= 3.13\times 10^{15}$, $3.13\times 10^{16}$, and $3.13\times 10^{17}$ hit the prey at $t'=0.5950$, 0.5885 and 0.5882, respectively. The stiffer barbs achieve faster ejection and stay on target, moving towards the prey. This trend demonstrates that increasing the barb’s stiffness improves ejection efficiency by reducing the time to reach its target.

**Fig. 16:**
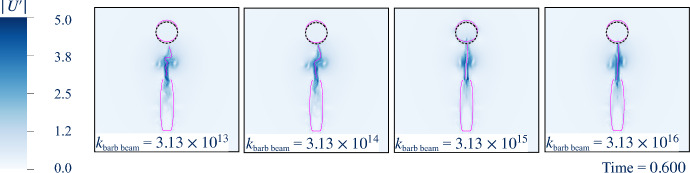
Dimensionless velocity magnitudes for $t'=0.6$ where the stiffness of the bottom three-quarters of the barb has been varied. The stiffness of the top 25% of the barb is set to $k'_\text {beam}=3.13\times 10^{16}$. The stiffnesses of the bottom three quarters of the barb are set to $k'_\text {beam}=3.13\times 10^{13}$, $3.13\times 10^{14}$, $3.13\times 10^{15}$, and $3.13\times 10^{16}$. Note that this means for the case where $k'_\text {beam}=3.13\times 10^{16}$, the entire barb has the same stiffness. The dimensionless magnitude of the velocity for each is given as a color map ranging from light blue (low) to navy (high). The magenta outlines represent the capsule, barb, and prey positions, with the initial prey location denoted by a dashed black circle.

Since nematocyst barbs are typically designed with a stiffer tip, we constructed barbs in which the bottom 50% or 75% was set to a range of stiffnesses, and the top section was fixed with $k'_\text {beam}=3.13\times 10^{16}$. Fig. 16 the dimensionless velocity magnitude of the flow field with the quarter barb, while Fig. 18 shows the dimensionless velocity magnitude of the flow around the half barb. In both cases, the stiffness of the ends was set to $k'_\text {beam}=3.13\times 10^{13}$, $3.13\times 10^{14}$, $3.13\times 10^{15}$, and $3.13\times 10^{16}$.

**Fig. 17:**
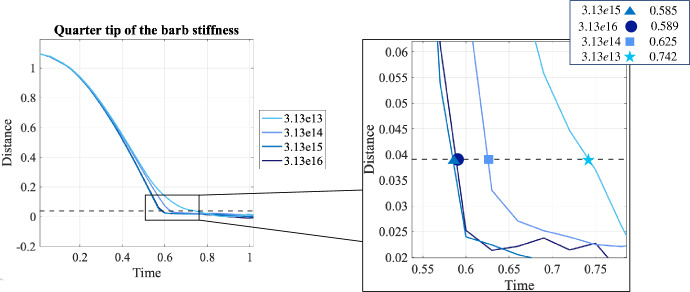
The shortest dimensionless distance between the barb and the closest point on the prey as a function of dimensionless time for the three-quarter barb case. The first 25% of the barb has the original stiffness $k'_\text {beam}= 3.13\times 10^{16}$, and the following three quarters has the stiffness set to $k'_\text {beam}= 3.13\times 10^{13},$ $3.13\times 10^{14},$ $3.13\times 10^{15}$, and $3.13\times 10^{16}$. Note that this means for the case where $k'_\text {beam}=3.13\times 10^{16}$, the entire barb has the same stiffness. The dashed line denotes the threshold value for the contact of $4\Delta x$. The inset shows a zoomed-in view of the dimensionless times to reach the prey for each variation of the barb’s stiffness.

As the stiffness is decreased for the lower three-quarters of the barb, the deformations of the barb are more significant (see Fig. 16). These deformations disturb the flow towards the end of the barb, and the flow field near the tip is relatively undisturbed. As such, the stiff tip does help in maintaining a relatively straight trajectory. In the cases of $k'_\text {beam}=3.13\times 10^{13}$ and $3.13\times 10^{14}$, the larger deformations of the end increase both the drag and the time required to reach the prey. Fig. 17 shows the dimensionless distance between the top of the barb and the closest point on the prey as the flexibility of the bottom three-quarters of the barb is varied. The inset shows the exact dimensionless time to hit for all four simulations. When the stiffness is set to $k'_\text {beam}=3.13\times 10^{13},$ $3.13\times 10^{14}$, $3.13\times 10^{15}$, and $3.13\times 10^{16}$, the dimensionless time to hit the prey is $t'=0.585$, 0.589, 0.625, and 0.742, respectively. In general, more flexible ends result in longer times to hit the prey.

**Fig. 18:**
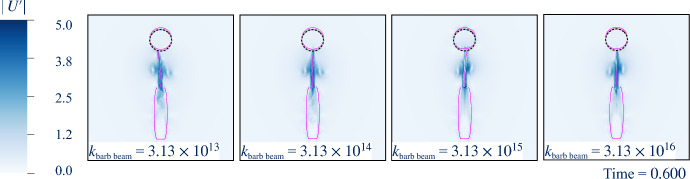
Dimensionless velocity magnitudes for $t'=0.6$ where the stiffness of the bottom half of the barb is varied. The stiffness of the top half of the barb is set to $k'_\text {beam}=3.13\times 10^{16}$. The stiffnesses of the bottom halves of the barbs are set to $k'_\text {beam}=3.13\times 10^{13}$, $3.13\times 10^{14}$, $3.13\times 10^{15}$, and $3.13\times 10^{16}$. Note that this means for the case where $k'_\text {beam}=3.13\times 10^{16}$, the entire barb has the same stiffness. The barb, capsule, and prey are represented in magenta, and the original prey position is shown as a black dashed circle. The magnitude of velocity is represented as a color map ranging from light blue (low) to navy (high).

**Fig. 19:**
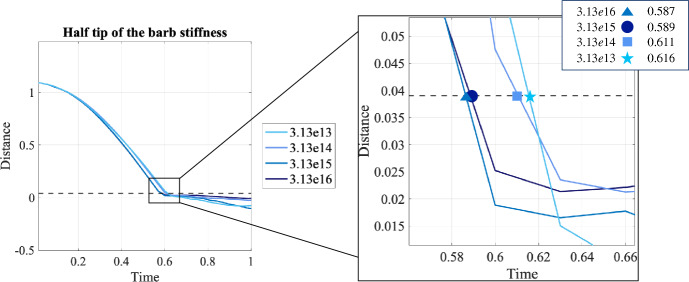
Dimensionless distance between the barb and prey as a function of time for simulations when the top half of the barb has a fixed flexibility and the flexibility of the bottom half is varied. The first 50% of the barb has the original stiffness $k'_\text {beam}= 3.13\times 10^{16}$, and the following half has the stiffness set to $k'_\text {beam}= 3.13\times 10^{13},$ $3.13\times 10^{14},$ $3.13\times 10^{15}$, and $3.13\times 10^{16}$. Note that this means for the case where $k'_\text {beam}=3.13\times 10^{16}$, the entire barb has the same stiffness. The dashed line denotes the threshold value for the contact of $4\Delta x$. The inset shows the dimensionless time to reach the prey for the three highest barb stiffness.

Fig. 18 shows the dimensionless velocity magnitude of the flow field when the top half of the barb has a fixed flexibility, and the flexibility of the bottom half is varied. When the stiffness of the lower half of the barb is smaller, we observe smaller deformations and faster times to reach the prey when compared to the equivalent three-quarter stiffness case. In other words, if a larger section of the barb is flexible, deformations are larger, and the time to hit the prey is longer. As we increase the stiffness of the bottom half of the barb, we observe smaller deformations, and the barbs travel further from the capsule. Fig. 19 shows the dimensionless distance between the top of the barb and the closest point on the prey as a function of dimensionless time for the half-barb cases. The inset shows the exact dimensionless times the barbs hit the prey. Note that the best and worst hit times are within 0.270 of each other. These results confirm that more flexible barbs result in larger deformations, disturbances in the fluid, and longer times to hit the prey.

## Discussion

Using a computational fluid dynamics model of extrusome ejection, we have found the following results: (1) the collapse of the capsule results in a large outflux of flow, imparting considerable forward momentum of the barb, (2) increasing *Re* provides only modest increases in the dimensionless distance the barb travels, (3) the relationship between *Re* and the dimensionless time to hit the prey is non-monotonic with intermediate *Re* barbs reaching the prey sooner, (4) larger percent volume changes decreased the dimensionless time it takes the barb to hit the prey, (5) smaller gap sizes increase the fluid and barb dimensionless velocity through the exit, decreasing the dimensionless time to hit the prey, and (6) more flexible barbs typically take longer to reach the prey or do not reach it at all.

Initially, we performed simulations across a range of *Re* with and without prey. The spatial parameters were estimated from Rosati and Modeo (2003), and the mechanical properties were empirically determined, ensuring minimal deformations of the boundaries, except in cases where flexibility was explored. The base case demonstrated the foundational dynamics of extrusome ejection, where the collapse of the capsule drives the internal fluid and the barb outward. Upon exiting the capsule, the barb is immersed within a jet of fluid whose width is inversely proportional to the *Re*. We found that there was little difference between the no-prey and prey cases except when the barb comes close to and contacts the prey.

The *Re* for all sets of simulations was varied from 0.287 to 1000, representing both the viscous-dominated and inertial-dominated cases. At high *Re* (e.g., 500 and 1000), the barb moves farther when no prey is present and on a more direct trajectory. When the prey is present, the high *Re* barbs make contact with the prey at $t'\approx 0.588$. Lower *Re* results in a wider jet and movement of the prey out of the way of the barb due to the large fluid boundary layer. When considering the distance between the barb and the prey, the *Re* results are non-monotonic as the barb at intermediate *Re* contacts the prey sooner. To compare these results to the case when the barb is initially farther from the prey, higher *Re* barbs do make contact with the prey sooner than lower *Re*. For the cases where $Re < 287$, the barb does not make contact with the prey at all. Overall, higher *Re* barbs show consistent success in prey contact even when starting distances increase, demonstrating their robustness to varied configurations.

To determine the effects of changing the volume ejected, we varied the initial minor axis of the capsule, *a*. The minor axis was set to 0.08, 0.16, 0.20, 0.22, and 0.24. This corresponds to changes in capsule volume of 55.7%, 71.5%, 75.9%, 77.6%, and 78.4%, respectively. Increasing the initial length of the minor axis inherently modifies the capsule geometry, increasing the structural deformation during ejection. There is a nearly linear relationship between the dimensionless time to hit the prey and the percent volume change; larger deformations of the capsule correlate with faster ejections and decreased time to hit the prey. Also, the disturbance in the surrounding fluid is larger for larger volume changes, suggesting an inefficient transfer of energy to the fluid. These results highlight the critical role of capsule geometry in optimizing energy transfer and ejection efficiency.

We varied the width of the capsule opening to determine how this parameter affected the ejection of the barb from the capsule. Typically, the exit region of an extrusome does not appreciably change in length, and the capsule does not fully collapse on itself. To model this geometry, we used a plate of length $2\epsilon$ to define the base of the capsule, and the capsule opening had the same dimension. We set this dimensionless distance to be 0.16, 0.20, 0.24, and 0.28. As the gap size decreased, the dimensionless time to hit the prey also decreased nearly linearly. The smallest gap size, $2\epsilon =0.16$, produced the strongest jet due to the smaller exit area. We note that the same volume of fluid ($ab \pi$) was ejected over the same amount of time for each gap length, so by conservation of mass, the fluid speed increased as the gap length decreased.

The effects of barb flexibility on its trajectory were explored by varying the bending stiffness of the bottom half of the barb and then the bottom three-quarters of the barb. When the stiffness of the barb was varied, larger values of $k_\text {beam}$ improved the alignment of the barb and reduced the time to hit the prey. Lower stiffness values failed to maintain straight trajectories, causing the barbs to ‘snake’ through the fluid. If changes in flexibility were restricted to the lower half of the barb, the effect would be smaller than that of changing the stiffness of the lower three-quarters of the barb. A stiff top of the barb allows for relatively straight trajectories toward the prey. The main effect of the flexible region on the trajectory was that relatively flexible ends generated large deformations that disturbed the fluid and generated more drag. Overall, these simulations emphasize the importance of stiffness in controlling localized barb deformations and the generation of drag.

In this computational study of extrusome ejection, we present a detailed analysis of the dynamics governing the interaction between fluid flow, capsule deformation, and the barb’s trajectory toward prey. By varying key parameters such as the Reynolds number, capsule geometry, and barb stiffness, we determined their effects on barb dynamics and the ability to hit a target. While the study was performed in two dimensions, it provides a foundation for extending the analysis to three-dimensional models, where the added complexity of 3D spatially varying pressures, velocities, and structural deformations could yield significant insights into the full dynamics of ejection.

Our focus on the capsule collapse and resulting fluid jet provided valuable insights into how these factors drive the motion of the barb. However, it did not include the complexities of contact or puncture dynamics. Future studies should explore how the barb interacts with prey tissues, accounting for both barb and prey deformation, the fluid dynamics involved in venom delivery, and the localized forces generated during puncture. These processes are relevant in other biological contexts, such as snake venom puncture and delivery studied in Young et al. (2018). A general energy-balance model for biological puncture events was proposed to analyze commonalities across puncture systems in Zhang and Anderson (2022). Their mathematical framework outlines how the geometry and material properties of a puncture tool govern the energy required to breach a flexible material boundary, which directly complements our findings on how barb stiffness affects prey contact.

Placing our results in this broader context, the capsule collapse we model sets the upstream conditions, barb velocity, alignment, and the fluid jet around it, that any downstream puncture or injection step must then work with. The energy-balance framework of Zhang and Anderson (2022) shows that whether a sharp tool successfully breaches a flexible boundary depends on both the kinetic energy delivered and the geometry of the tool tip, and our finding that flexible barbs lose energy to fluid drag and lateral deformation directly limits the energy available for puncture. Follow-up work has shown that the sensitivity of puncture performance to tip sharpness decreases at higher puncture speeds (Zhang and Anderson 2023), and that structural curvature of the puncture tool has surprisingly little effect on performance (Zhang et al. 2024). Both findings are consistent with our use of a straight barb and suggest that, in the inertial regime our model targets, the dominant constraints on a successful strike come from the fluid jet and barb deflection rather than from fine details of tip geometry. The reptilian envenomation work of Young et al. (2018) highlights a related point at a very different scale: even after a delivery structure is in place, the hydrodynamics of the venom itself govern how the payload is transported into the target. Together with our results, this suggests extrusome performance cannot be evaluated by ejection speed alone, the full sequence of capsule collapse, barb transit, puncture, and fluid delivery is mechanically coupled, and tradeoffs at one stage constrain the others.

Our idealized model of extrusomes, including simplified capsule and barb properties, was designed to establish a baseline for understanding the core ejection mechanisms at the microscale. Rather than focusing on the diversity of chemical and physical processes found across various extrusomes (Hausmann 1978; David et al. 2008; Hausmann and Mignot 1975), our model provides general insight into the mechanical processes involved in the fluid-structure interactions leading to projectile-based motions. Results may also provide insights into the evolutionary pressures shaping extrusome design. In Gavelis et al. (2017), the authors found that non-homologous ‘nematocysts’ found in predatory dinoflagellates functionally mirror those of multicellular cnidarians and suggest convergent evolution of extrusomes in these systems. Our fluid-structure interaction simulations suggest that, despite completely separate evolutionary lineages, these micro-scale weaponry systems are strictly bound by the same underlying physical principles.

The diversity of extrusome morphologies across protists is striking and largely independent of the cnidarian lineage. Ciliates alone produce a wide range of extrusive organelles, trichocysts, mucocysts, toxicysts, and rhabdocysts, that differ in shape, discharge mechanism, and ecological role (Rosati and Modeo 2003; Hausmann 1978). Even within the trichocyst-like category, new variants continue to be characterized, including the recently described protrichocyst, which couples a millisecond-scale, three-stage mechanical ejection to a toxicyst-like chemical release (Dong et al. 2025). Some, such as *Paramecium* trichocysts, rely on rapid mechanical unfolding of a protein lattice, while others, such as dinoflagellate nematocysts, achieve cnidarian-like complexity through entirely separate evolutionary routes (Gavelis et al. 2017). Despite these differences in molecular machinery, the structures all share the same basic functional problem: a stored configuration must be rapidly converted into directed motion of a projectile through a viscous fluid. Our results suggest that this shared physics constrains what is achievable at the cellular scale, regardless of how the energy is initially stored or released. The non-monotonic dependence of contact time on *Re*, the sensitivity to gap geometry, and the penalty paid by flexible barbs are features of the fluid-structure interaction, not of any one biochemical mechanism. This may help explain why such different lineages converge on broadly similar performance envelopes.

This perspective also connects to broader work on power-amplified biological motion. Systems ranging from mantis shrimp strikes to fungal ballistospores to extrusome discharge all sit at the intersection of stored elastic energy, geometric amplification, and fluid resistance (Ilton et al. 2018; Liu et al. 2017). Our model adds a microscale, fluid-coupled case to this literature and shows that, at Reynolds numbers near unity and below, the fluid boundary layer becomes a dominant constraint on whether the projectile actually reaches its target. Evolutionarily, this may favor either steeper increases in Reynolds number (through faster discharge) or alternative strategies, such as a longer extended tubule or a tethered prey-capture step, that bypass the boundary-layer problem altogether. To better resolve the diversity of real-world extrusomes, future work should expand the modeling to include variations in ejection processes, structural properties, and environmental conditions.

In general, this study expands on previous work in modeling the fluid dynamics of extrusome discharge (Hamlet et al. 2020), including the multi-barb extension in Harrison et al. (2025), and lays the groundwork for future research aimed at a more comprehensive understanding of the biomechanics of extrusome function. By incorporating three-dimensional dynamics, contact mechanics, and biological diversity, we can develop a more complete understanding of these structures and their functions.

## Data Availability

Data sets generated during the current study are available from the corresponding author on reasonable request.
